# Efficacy of manipulation for non-specific neck pain of recent onset: design of a randomised controlled trial

**DOI:** 10.1186/1471-2474-8-18

**Published:** 2007-02-26

**Authors:** Andrew M Leaver, Kathryn M Refshauge, Christopher G Maher, Jane Latimer, Rob D Herbert, Gwendolen Jull, James H McAuley

**Affiliations:** 1 Back Pain Research Group, Discipline of Physiotherapy, University of Sydney, PO Box 170 Lidcombe NSW 1825 Australia; 2 Division of Physiotherapy, University of Queensland, Brisbane Qld 4072 Australia

## Abstract

**Background:**

Manipulation is a common treatment for non-specific neck pain. Neck manipulation, unlike gentler forms of manual therapy such as mobilisation, is associated with a small risk of serious neurovascular injury and can result in stroke or death. It is thought however, that neck manipulation provides better results than mobilisation where clinically indicated. There is long standing and vigorous debate both within and between the professions that use neck manipulation as well as the wider scientific community as to whether neck manipulation potentially does more harm than good. The primary aim of this study is to determine whether neck manipulation provides more rapid resolution of an episode of neck pain than mobilisation.

**Methods/Design:**

182 participants with acute and sub-acute neck pain will be recruited from physiotherapy, chiropractic and osteopathy practices in Sydney, Australia. Participants will be randomly allocated to treatment with either manipulation or mobilisation. Randomisation will occur after the treating practitioner decides that manipulation is an appropriate treatment for the individual participant. Both groups will receive at least 4 treatments over 2 weeks. The primary outcome is number of days taken to recover from the episode of neck pain. Cox regression will be used to compare survival curves for time to recovery for the manipulation and mobilisation treatment groups.

**Discussion:**

This paper presents the rationale and design of a randomised controlled trial to compare the effectiveness of neck manipulation and neck mobilisation for acute and subacute neck pain.

## Background

Manual techniques are routinely used in the management of non-specific neck pain and appear to provide effective pain relief for at least some neck conditions [1-5]. These techniques include manipulation, a high velocity thrust directed at the joints of the spine, and mobilisation techniques that do not involve a high velocity thrust. Manipulation is associated with a small risk of serious cerebrovascular injury [6,7], whereas mobilisation is generally considered to be a safer technique [8]. However in clinical situations where manipulation is indicated it is thought to provide better results than mobilisation.

Both manipulation and mobilisation are more effective in relieving chronic neck pain than general practitioner care [3,9] or no treatment controls[2]. Few trials have investigated manual therapy for acute neck pain [1] or compared the effectiveness of manipulation and mobilisation[2]. There is some evidence that a single manipulation provides greater improvement in neck pain and range of motion, than mobilisation when these outcomes are measured immediately following a treatment [10,11]. It is not known however, if manipulation leads to more rapid or more complete recovery from an episode of recent onset neck pain than safer manual therapy techniques.

Neck manipulation is an independent risk factor for vertebrobasilar stroke in young adults [6,12]. The proposed mechanism of injury is dissection of the intima or media lamina of the vertebral artery [13]. Compromise of vertebrobasilar circulation can result in mild transient symptoms such as dizziness or loss of balance, or in more serious cases "locked in" syndrome, Wallenberg syndrome or death [7,14,15]. Estimates of incidence of serious complications from neck manipulation are imprecise, ranging between 1 serious complication for every 1,000,000 treatments [16,17] and 1 for every 10,000 treatments [18]. It is generally accepted that the risks might be higher [14,17,19,20] because there are no mandatory reporting requirements or standard systems for reporting manipulation accidents. Although cerebrovascular complications from neck mobilisation have been reported, these are mostly of a transient and minor nature [21,22]. The only exception is one recorded case of stroke, following vigorous rotation mobilisation [22].

There is long standing and vigorous debate both within and between the professions that use neck manipulation as well as the wider medical community as to whether neck manipulation does more harm than good[23]. Manipulation has potential benefits that include hastening of recovery of symptoms and restoration of normal function in the early stages of an episode of neck pain. We will conduct a randomised controlled trial to determine whether neck manipulation provides faster resolution of symptoms than mobilisation in patients with an episode of acute or subacute non-specific neck pain. It is hypothesised that an acute episode of neck pain will resolve more rapidly when treated with manipulation than with mobilisation.

## Methods/Design

A randomised controlled trial with two treatment arms will be conducted at private physiotherapy, chiropractic and osteopathic clinics in Sydney, Australia. Participants with neck pain of recent onset will be randomised to receive treatment with either manipulation or mobilisation. Randomisation will occur at the treatment session at which the treating practitioner decides that manipulation is an appropriate treatment. Ethics approval has been granted by The University of Sydney Human Research Ethics Committee. Consent will be obtained from each participant prior to entry into the trial.

### Study Sample

One hundred and eighty-two participants will be recruited from among patients presenting for physiotherapy, chiropractic or osteopathic treatment of acute or subacute neck pain. Patients who satisfy the inclusion criteria will be invited to participate in the study.

### Inclusion criteria

The aim of this trial is to establish the effectiveness of manipulation compared with mobilisation for conditions that are treated with manipulation in contemporary practice and are likely to benefit from manipulation. For this reason participants are only eligible for inclusion in the trial if the treating practitioner decides that manipulation would be an appropriate treatment for the individual patient. Patients for whom manipulation is thought not to be indicated will be excluded by the practitioner. In these cases the practitioner is asked to provide a reason why they consider manipulation to be inappropriate in the circumstances.

Participants entering the trial must be aged between 18 and 70 years and have a primary complaint of non-specific neck pain that is located at least partly in the area defined by Merskey [24]

*anywhere within the region bounded superiorly by the superior nuchal line, inferiorly by an imaginary line through the tip of the first thoracic spinous process and laterally by sagittal planes tangential to the lateral borders of the neck*. p 11

The episode of neck pain must be of less than 3 months duration and preceded by at least one month without neck pain. The pain must be of sufficient intensity (greater than 2 out of 10 on a numerical pain scale) to permit a clinically worthwhile effect to be demonstrated.

### Exclusion criteria

Participants will be excluded if their neck symptoms are related to a motor vehicle accident or significant trauma; if their condition involves predominantly arm symptoms; if there are signs of serious pathology such as malignancy, infection, inflammatory disorder or fracture; if there are signs of cervical spinal cord compromise (determined by the presence of any of the following signs; diffuse sensory abnormality, diffuse weakness, hyperreflexia or presence of clonus) or radiculopathy (determined by the presence of 2 of the following signs; dermatomal sensory abnormality, myotomal weakness or diminished/absent tendon jerk reflexes) or if they have undergone neck surgery in the previous 12 months.

A record will be kept of the number of subjects excluded from the study as well as those who are eligible for inclusion and choose not to participate. Reasons for not participating will be sought.

### Baseline measurements

Baseline data will be collected at the initial appointment about the demographic and clinical characteristics of participants. Socio-demographic data will be collected using items from the Australian Census 2001 [25] and using standard classification of employment [26] and education status [27]

We will collect measures of pain [28] (average pain over the last 24 hours rated on a 0–10 numerical rating scale) neck-related disability [29], patient-specific disability [30] and quality of life [31] when patients first present for treatment. The pain, disability and quality of life measures will be assessed again immediately prior to randomisation as baseline outcome measures for the trial.

### Treatment allocation

Current neck manipulation guidelines recommend that initial treatments be conservative and the effects of treatment carefully monitored prior to treatment with neck manipulation [8]. Participants will be randomised to a treatment group only after the treating practitioner has established that neck manipulation is the treatment of choice in the circumstances. Late randomisation is a feature of good trial design [32] and in this case will ensure that the trial reflects current clinical practice. In the period prior to randomisation participants will receive treatment that is consistent with evidence-based guidelines for the treatment of acute neck pain [33].

A statistician who is not involved in subject recruitment or data collection will produce consecutively numbered sealed opaque envelopes containing the treatment allocation for each patient. The randomisation sequence will contain equal numbers of subjects in each group but will be otherwise unrestricted.

### Interventions

Assessment and treatment will be conducted by experienced manipulative physiotherapists, chiropractors and osteopaths. Participating physiotherapists will have postgraduate university qualifications in manipulative physiotherapy and at least 2 years post graduate experience. Participating chiropractors and osteopaths will have a university chiropractic or osteopathic degree and at least 2 years of clinical experience.

Participants will be allocated to one of two treatment arms.

*Manipulation Group: *a course of manual treatment consisting of high velocity, low amplitude movements applied to the cervical spine by the therapist. Participants will receive at least 4 treatments over 2 weeks.

*Mobilisation Group: *a course of manual treatment consisting of low velocity oscillatory movements applied to the cervical sine by the therapist. Participants will receive at least 4 treatments over 2 weeks.

Treatment may be discontinued prior to the fourth treatment, in either of the treatment arms if the participant completely recovers or if they experience a serious adverse response to treatment. Within these guidelines the number and type of manipulation and mobilisation techniques will be determined by the practitioner. The type of manipulative or mobilising technique will not be standardised, as this might result in inappropriate treatment for some patients. The practitioner will keep a record of the number and type of techniques used. The intervention period will be up to 2 weeks.

Clinical practice guidelines recommend that manipulation and mobilisation be used as part of a multimodal management strategy incorporating other effective treatments rather than as stand-alone techniques [34]. All participants may therefore receive other evidence-based treatments including advice, reassurance, encouragement to resume usual activities, and will be asked to continue with any exercise regimen that they had previously commenced. The use of manipulation or mobilisation of the thoracic or lumbar regions of the spine will not be constrained in either group. Treatment with a combination of neck manipulation and mobilisation techniques, as sometimes occurs in clinical practice will not be permitted for either group.

During the intervention period both groups will be asked to refrain from seeking other treatment but will be permitted to continue pain medication. Participants will be asked to keep a record of any additional treatment received, the type of treatment, the date of this treatment and its effects. Participants will not be discouraged from seeking further treatment at the conclusion of the 2-week trial period, but will be asked to keep a record of any further treatment received as for the trial period.

### Outcome measures

#### Primary outcome

Outcome measures will be recorded by a researcher who is blinded to subject allocation. The primary outcome measure is the number of days taken to recover from neck pain. Participants will be considered to have recovered when they rate pain intensity as <1/10 for more than 7 consecutive days. For the purposes of analysis the recovery will be determined as the first of those 7 days.

Participants will be asked to keep diaries of pain intensity. They will record average pain over each 24 hour period on a 0–10 scale. Participants will be asked to complete this record on a daily basis for the first 4 weeks, then weekly for the next 4 weeks. Participants will be contacted by telephone on a weekly basis for the first 4 weeks to encourage them to complete their diaries and to collect diary data. Pain scores for the next 2 months will be collected by telephone at monthly intervals.

#### Secondary outcomes

The time to recovery from neck-related disability will be measured using a single item disability scale modified from the MOS-SF36 survey [35]. Participants will record in their diary the degree to which neck pain interfered with normal daily activities on a 5 point scale with responses ranging from "did not interfere at all" to "interfered extremely" with daily activities. Recovery is defined as a rating of "did not interfere at all" on this scale. Participants will be contacted by telephone on a weekly basis for the first 4 weeks to monitor compliance with completing the diary and to collect diary data. Disability scores will be collected by telephone at monthly intervals for the next 2 months.

A neck-specific measure of disability [29] and a patient-specific measure of disability [30] will be completed at the 4 week and 12 week phone interview. Global perceived effect of treatment [36] will be recorded at the 2 week and 12 week phone interview. Participants will be questioned at the 12 week phone interview about the frequency and duration of neck pain after recovery to determine the incidence of recurrent and new episodes of neck pain. As there are no accepted definitions of recurrence of an episode of neck pain, definitions proposed for recurrent episodes of low back pain will be used [37]. A new episode of neck pain is defined as an episode of neck pain lasting at least 24 hours preceded by at least one month without neck pain. A recurrence is defined as pain of at least 24 hours duration within one month of recovery. A record will be kept of the number of days lost from work due to neck pain over the 3-month period [36].

The participants' perception of the treatment credibility [38] will be recorded in the participant diary after the first treatment. The number and type of treatments provided in the period between the initial physiotherapy or chiropractic appointment and randomisation will be recorded by the practitioner after randomisation.

### Adverse Events

Information about adverse events and side effects will be collected by the treating practitioner after each treatment session using an open ended question and by the assessor at each telephone follow-up using an open ended question and using a checklist of known side effects of manual therapy [39]. Participants will also be asked at the final 3-month follow-up about any side effects of treatment. In the unlikely event of a major complication such as stroke the emergency protocol recommended by the Australian Physiotherapy Association [8] will be followed. Potentially serious events will be immediately referred to the patient's medical practitioner and/or the closest hospital emergency department.

### Data analysis

Data will be analysed by a statistician who will be blinded to group allocation. The primary analysis will be by intention-to-treat. Cox regression will be used to compare survival curves (time to recovery from pain) of manipulation and mobilisation groups, provided the assumption of proportional hazards is met.

The between-group differences of continuously distributed secondary outcomes will be examined using parametric and non-parametric procedures as indicated. Potential predictors of treatment outcome will be evaluated by examining treatment × predictor interactions in linear, logistic and survival models.

### Sample size calculations

The sample size of 182 participants gives an 80% probability of detecting a reduction in median survival time from 21 to 12 days, or equivalently from 35 to 20 days assuming alpha = 0.05.

## Discussion

This paper outlines the rationale and design for a randomised controlled trial that compares the effectiveness of manipulation for recent onset neck pain with a safer alternative, mobilisation. Improved knowledge about relative benefits of neck manipulation will better inform clinical decision making and policy development in this controversial field of clinical practice

## Competing interests

The author(s) declare that they have no competing interests.

## Authors' contributions

AL, KR, CM, JL, RH, GJ and JM were responsible for the design of the study. All authors read and approved the final manuscript.

## Pre-publication history

The pre-publication history for this paper can be accessed here:


